# High-Resolution Microfluidic Paper-Based Analytical Devices for Sub-Microliter Sample Analysis

**DOI:** 10.3390/mi7050080

**Published:** 2016-05-02

**Authors:** Keisuke Tenda, Riki Ota, Kentaro Yamada, Terence G. Henares, Koji Suzuki, Daniel Citterio

**Affiliations:** Department of Applied Chemistry, Keio University, 3-14-1 Hiyoshi, Kohoku-ku, Yokohama 223-8522, Kanagawa, Japan; kei-54ppp@keio.jp (K.T.); ricky@keio.jp (R.O.); ymkn.z3@keio.jp (K.Y.); tghenares@gmail.com (T.G.H.); suzuki@applc.keio.ac.jp (K.S.)

**Keywords:** µPAD, wax printing, inkjet printing, colorimetry, protein assay

## Abstract

This work demonstrates the fabrication of microfluidic paper-based analytical devices (µPADs) suitable for the analysis of sub-microliter sample volumes. The wax-printing approach widely used for the patterning of paper substrates has been adapted to obtain high-resolution microfluidic structures patterned in filter paper. This has been achieved by replacing the hot plate heating method conventionally used to melt printed wax features into paper by simple hot lamination. This patterning technique, in combination with the consideration of device geometry and the influence of cellulose fiber direction in filter paper, led to a model µPAD design with four microfluidic channels that can be filled with as low as 0.5 µL of liquid. Finally, the application to a colorimetric model assay targeting total protein concentrations is shown. Calibration curves for human serum albumin (HSA) were recorded from sub-microliter samples (0.8 µL), with tolerance against ±0.1 µL variations in the applied liquid volume.

## 1. Introduction

In recent years, microfluidic paper-based analytical devices, commonly referred to as µPADs, have gained a lot of attention as potential alternative tools for various analytical tasks. The attractive features of µPADs are to a large extent related to the use of paper as the substrate material and include low cost, easy disposability, as well as external power-free sample transport driven by capillary forces. In addition, µPADs are generally easy to fabricate, user-friendly, and offer simple readouts of analytical assay results. The continuously growing interest in this type of paper-based devices is demonstrated by the fact that more than 100 research articles including the term “μPADs” have been published during the 2014–2015 period alone [1]. The advantageous characteristics described above have led to the application of µPADs especially for biomedical and environmental analysis, as well as for the monitoring of food and beverage contamination [2,3,4,5,6]. 

One disadvantage of μPADs in general, however, is that they mostly require larger sample volumes compared to conventional microfluidic devices based on glass or polymeric substrates [7,8,9]. This is due to the mostly larger dimensions of microfluidic structures patterned on paper substrates and the fact that µPADs are generally open systems prone to evaporation of sample fluids during the slow wicking process in paper microfluidic channels. Although there are many types of practical applications where this is not an issue, it will pose restrictions on the use of μPADs with samples that are only available in very small quantities, such as tear fluid [10] or blood plasma obtained from a finger prick blood sample passed through a blood cell retaining filter [11]. Sample volumes in the sub-microliter order are not sufficient to wet out the microfluidic structure of µPADs and to reach the zone where signal creation takes place.

Since the first report on photolithographically patterned µPADs by the Whitesides group in 2007 [12], a large number of more low cost, simple, and rapid alternative patterning methods for paper substrates has been developed. Major approaches include wax printing, inkjet printing, flexographic printing, knife cutting, laser cutting, and stamping [2]. Microfluidic channels with widths down to the order of 300 µm have been achieved. Among all of these methods, wax printing has evolved into the most widely used patterning technique, because of its ease and flexibility of computer-based pattern design (Adobe Illustrator, Microsoft PowerPoint, *etc.*), small number of process steps (printing followed by heating), and suitability for mass production at low cost [13,14]. The method relies on the printing of a solid wax-based ink in a pattern outlining the desired microfluidic structure, followed by a heating step wherein the wax is melted to penetrate into the paper, finally resulting in the formation of water impermeable hydrophobic barriers throughout the thickness of the paper.

However, fabricating highly resolved microfluidic structures meeting the requirements for sub-microliter sample volume assays by this technique remains challenging. One main issue is that the printed wax does not only vertically penetrate into the paper substrate to form the desired hydrophobic barriers, but at the same time also undergoes horizontal diffusion, leading to an inevitable blurring of the originally sharp printed structures [13,15]. This drawback prevents the applicability of the simplest paper patterning method for the fabrication of µPADs applicable to assays with very small sample volumes. Although a paper-based device obtained by a craft punch technique suitable for the handling of sub-microliter sample volumes has been reported, no sample liquid transport in a microfluidic channel is involved, and samples have to be applied directly to the detection zone [16]. 

The goal of the present work is the fabrication of µPADs enabling the distribution of a sub-microliter sample from a single inlet into multiple detection zones. Focus is set on a hot laminator as the post-print heating method, since one cause of horizontal wax diffusion lies in the use of hot plates or ovens conventionally chosen to melt the printed wax into the paper substrate. If a laminator is used in combination with lamination films, the evaporation of sample fluid can be prevented at the same time. Therefore, this approach is expected to eliminate the two major causes for the requirement of larger sample volumes with µPAD assays compared to conventional microfluidics mentioned above. Finally, the closed space produced by lamination of the entire device contributes to a more controlled sample uptake by the μPAD, increasing the tolerance in the amount of applied sample volume by the end user. This is important when working with minuscule sample volumes, where precise pipetting is more challenging. Although the combination of wax printing with a hot laminator has been used before [15,17], high-resolution µPADs for the analysis of sub-microliter samples, to the best of our knowledge, have not been investigated.

## 2. Materials and Methods

### 2.1. Reagents and Equipment 

All reagents were used as received without further purification. Citric acid and tetrabromophenol blue (TBPB) were purchased from Sigma-Aldrich (St. Louis, MO, USA). The phospholipid 1,2-dioleoyl-*sn*-glycero-3-phosphocholine was obtained from TCI (Tokyo, Japan), and ethanol (for HPLC 99.5%) from Kanto Chemical (Tokyo, Japan) and *N*-(2-hydroxyethyl)-1-piperazine ethanesulfonic acid (HEPES) was purchased from Rikaken (Aichi, Japan). Sodium hydrogen phosphate, sodium chloride, potassium chloride, calcium chloride, magnesium chloride hexahydrate, and human serum albumin (HSA) were purchased from Wako Pure Chemical Industries (Osaka, Japan). Whatman No.1 filter paper (460 mm × 570 mm) was purchased from GE Healthcare Life Sciences (Buckinghamshire, UK). A Xerox ColorQube 8570 wax printer (Xerox, Norwalk, CT, USA) was used to print microfluidic structures designed in Adobe Illustrator CC software. The colorimetric assay reagent was deposited onto the paper devices using an iP2700 inkjet printer (Canon, Tokyo, Japan), where the original ink cartridges have been cut open and cleaned, and the sponges removed. Hot lamination films (thickness 150 µm) were obtained from Jointex (Tokyo, Japan). A Silhouette Cameo electronic knife blade cutting device (Silhouette, Lehi, UT, USA) in double cutting mode was used to cut sample inlet holes into the top lamination film layer. Hot lamination was performed on a QHE325 laminator (Meiko Shokai, Tokyo, Japan). The instrument used only allows for the optimization of substrate thickness and feeding speed. In all experiments, substrate thickness was set to the maximum available value (the “250 µm” position) and the “fast” feeding speed (experimentally measured as 39.4 ± 0.4 cm/min; *n* = 8). According to information provided by the instrument manufacturer, the maximum thickness setting corresponds to maximum heating temperature, but temperature values are not provided. The hot plate used in comparison experiments (Nissin NHS-450ND) was obtained from Nissinrika (Tokyo, Japan). A 9000F MARK II color scanner (Canon, Tokyo, Japan) was used to acquire colorimetric data, and the color intensity was measured by the Image J color image analysis software (NIH, Bethesda, MD, USA). A DVM2500 digital microscope (Leica, Wetzlar, Germany) was used for measuring the widths of hydrophobic barriers and microfluidic channels.

### 2.2. Device Fabrication 

**Figure 1:**
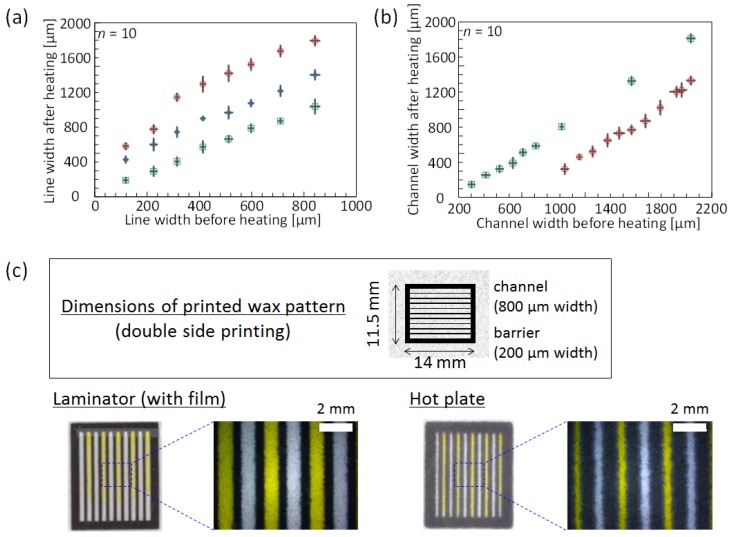
Comparison of resolution achieved for melting of printed wax into paper substrates by hot plate or hot lamination. (**a**) Wax line widths observed directly after printing and after heating with hot plate (red circles), hot laminator without top side lamination (blue diamonds), and hot laminator with full lamination (green squares) (mean value ± 1σ); (**b**) Microfluidic channel widths after heating by hot plate (red circles) or hot laminator with full lamination (green squares) (mean value ± 1σ); (**c**) Dimensions and photographs of 10 parallel microfluidic channels after lamination or hot plate (150 °C for 15 s) treatment. Channels visualized by application of colored aqueous solution.

In general, identical patterns of wax were printed in alignment on both sides of the filter paper substrate cut into A4 size before use. An exception are the experiments described in Figure S1 of the Supplementary Materials, where patterns were only printed on one side of the paper. After printing, the wax-modified paper substrate was placed between the two layers of the laminate film, with 0.85 mm diameter holes cut out of the top layer for the sample inlet if required. The aligned devices were passed through the hot laminator twice in order to melt the printed wax into the paper. Only for comparison experiments discussed below Figure 1, wax was melted into paper substrates by hot lamination without laminate film coverage on the top side. In this case, the wax-modified side of the paper was covered with aluminum foil to prevent the wax from contaminating the rollers of the hot laminator, while the back side of the paper was in contact with laminate film. Alternatively, the wax was melted into the paper by placing it on the hot plate at 150 °C for 180 s. A solution of 0.5 wt % of acid yellow was used to visualize the microfluidic structures in all cases.

For devices used in HSA assays, the colorimetric reagent TBPB was dissolved in 95% (*v*/*v*) aqueous ethanol at a concentration of 3.3 mM and mixed with citric acid buffer (pH 4.00, 0.10 M) in a 1:1 ratio. This reagent ink was filled into the black ink cartridge of the inkjet printer and deposited before hot lamination with the printer in black-and-white mode (R, G, and B values set to 0) in 20 printing cycles as a rectangle with a height of 1.4 mm covering all of the detection areas.

### 2.3. Human Serum Albumin Assay 

HSA standard solutions of various concentrations (0, 2, 4, 6, 7, 8, 9, 10 mg/mL) were prepared in a HEPES buffer (pH 7.40, 10 mM) with a background of 150 mM NaCl, 20 mM KCl, 1.0 mM CaCl_2_, 0.6 mM MgCl_2_, and 0.36 mM phospholipid, simulating the composition of human tear fluid [18,19]. The standard solutions (0.7–0.9 μL) were pipetted onto the sample inlet port of the µPADs. After drying, the devices were scanned, and the color intensities measured. Throughout this work, the mean red (R) intensities obtained from the four detection areas were used as the quantitative signal.

## 3. Results and Discussion

### 3.1. Heating Method of Melting Printed Wax into Paper Substrates

At first, it was experimentally demonstrated in terms of the sharpness and resolution of patterned features that hot lamination offers advantages over a hot plate in melting printed wax into filter paper substrates. For this purpose, black wax lines (100–900 µm wide, in increments of 100 µm) were printed on the top side of the filter paper substrate. Similarly, two parallel lines (400 µm wide) were printed with various gaps (laminator: 300–2000 µm, hot plate: 1000–2000 µm) for the comparison of formed hydrophilic channel widths. As previously mentioned, horizontal wax diffusion inevitably reduces the resolution of printed patterns. However, Figure 1a demonstrates that the line widths after heating by a hot laminator are smaller than those obtained by hot plate treatment, and are even further reduced by full lamination (film thickness of 150 µm). Accordingly, decreased horizontal diffusion by hot lamination compared to plate heating also results in wider hydrophilic channels for patterns printed with equal line distances (Figure 1b). Patterning of microfluidic structures by hot lamination results in features with visibly higher resolution, as demonstrated by the 10 parallel microchannels in Figure 1c. The better resolution is attributed to the pressure applied through the hot rollers during the heating step from both sides of the paper, which results in not only shorter heating times but also an enhanced vertical penetration of wax over horizontal diffusion compared to the hot plate-based method. This assumption is experimentally supported by the fact that wax lines melted into paper by hot plate treatment result in significantly narrower widths in the case of using compressed filter paper (for details, please refer to the Supplementary Materials), compared to untreated filter paper (Figure S1). Figure S1 also demonstrates that shorter heating time is helpful in creating thinner wax lines. It is postulated that the closer proximity between vertically compressed cellulose fibers leads to dominant diffusion in the paper thickness direction, while at the same time the horizontal flow of wax is reduced. The actual significant decrease in thickness associated with the compression of the paper substrate in the hot laminator is shown in Figure S2. It was further evaluated whether similar effects could be achieved without the use of a hot laminator by applying pressure during the hot plate treatment of wax-printed filter paper. Figure S1 shows that, even if heating time is shortened to 30 s under 22 kg of uniform pressure, a 300-μm printed wax line widens to 869 ± 32 μm (*n* = 5). This value is much larger than in the case of the full lamination-based method (405 ± 47 μm; *n* = 10) (Figure 1a). Shortening the heating time further and placing heavier weights on the hot plate would be detrimental to experimental simplicity. It can be concluded that the conventional hot plate treatment cannot compete with the presented hot lamination method in terms of achievable resolution.

The horizontal error bars in Figure 1a,b, indicating the standard deviations in line and channel widths before heating, are an indication of the achievable precision of the wax printing method itself and of variations in line widths caused by the inherent roughness of the filter paper used.

### 3.2. Device Design and Optimization

In order to achieve high-resolution μPADs, which can be used with sample volumes of no more than 1.0 μL, a variety of factors had to be considered. Besides the more obvious need to create the narrowest possible reliable hydrophobic barriers and channel widths for decreasing the overall size of the system, factors such as the influence of cellulose fiber direction and overall device geometry on sample wicking were evaluated before deciding on an appropriate design model. Throughout this work, microfluidic structures were created by printing identical patterns on both sides of the filter paper. This is in contrast to previous reports on wax-printed µPADs, where patterns are printed on one surface of the substrate and allowed to penetrate throughout the thickness of the paper during the post-print heating process. The double-sided patterning approach allows the printing of narrower line features, since wax penetrates into the paper from two directions, reducing the amount of ink required on each surface of the paper. It should be noted that the printing of identical patterns in full alignment on opposite paper surfaces calls for careful handling. First, if paper substrates are not precut, size-reproducible cutting is required, but can be readily achieved by using a paper cutting device. Second, the absolute position of features printed on the two paper surfaces needs to be fine-tuned in the graphic software, since actually printed patterns subtly go out of alignment upon printing on both sides of a paper sheet. Shifts need to be applied particularly perpendicular to the paper feeding direction. Thus, a trial-and-error approach is initially inevitable to perfectly match wax patterns on both sides of the paper substrates, since the required shifts are dependent on the position along the paper feeding direction. Finally, attention must be paid to reversible paper feeding into the printer, which is best achieved by relying on the manual feed tray, where the paper can be manually placed and fixed.

#### 3.2.1. Influence of Cellulose Fiber Direction

**Figure 2:**
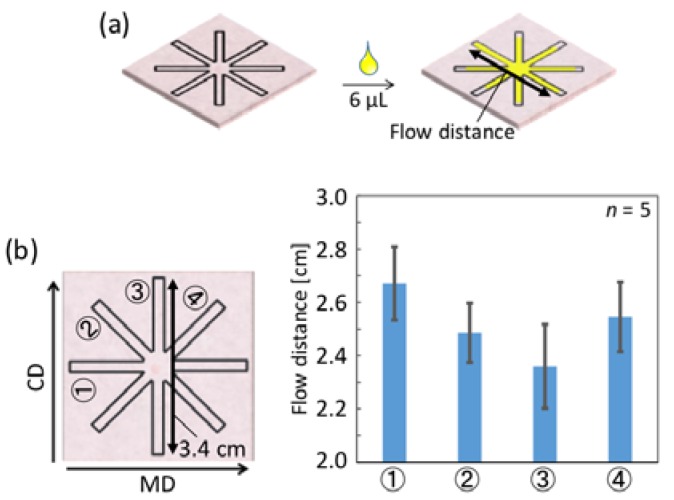
(**a**) Schematic representation of the evaluation of the influence of cellulose fiber direction on sample wicking in patterned filter paper (channel width: 553 ± 31 µm (*n* = 20) after lamination). The flow distances are measured as indicated by the arrow; (**b**) Quantitative results averaged for 5 independently fabricated devices (mean value ± 1σ). Circled numbers indicate the respective flow direction.

During the general paper fabrication process, cellulose fibers become dominantly aligned parallel to a specific direction, known as the machine direction (MD), in contrast to the perpendicular cross direction (CD) [20]. In the context of µPADs and in particular with narrow flow channels, this fiber direction is expected to affect the sample wicking process. To realize µPADs for the analysis of sub-microliter samples, the influence of different fiber directions has to be taken into account. For this reason, the effects of fiber directions were experimentally evaluated by measuring the maximal sample flow distance of a colored aqueous solution in differently orientated microfluidic channels (Figure 2a). The corresponding results are shown in Figure 2b.

Figure 2b clearly demonstrates that significantly longer flow distances are achieved in microfluidic channels aligned to the machine direction (① in Figure 2b) with parallel orientation of the cellulose fibers, compared to the cross direction (③ in Figure 2b), where flow occurs perpendicular to the cellulose fiber orientation. Channels arranged in a 45° angle to MD or CD (② and ④ in Figure 2b) showed flow distances approximately between the two extremes. The minor differences observed for the two 45° oriented channels (② and ④), as well as the relatively large error bars, can be attributed to the fact that, in spite of the paper-machining-induced general trend of fiber orientation, random local variations do exist. These results confirm that the cellulose fiber direction in the paper substrate does affect sample flow distance. To further investigate the influence of fiber direction on sample fluid transport, the flow velocity of dye solutions has been measured in single channels of different orientation. As shown in Figure S3, fiber orientation does influence the wicking speed. Observed flow speeds decrease in the order of MD-oriented (③ in Figure S3b), 45° rotated (② in Figure S3b), and CD-oriented (① in Figure S3b) microfluidic channels. These effects have to be taken into account when designing a µPAD for small sample volumes, where the machine direction is the more suitable orientation for efficient sample liquid transport. As a consequence, all microfluidic channels described in the following sections have been patterned along MD, with the exception of circular shapes, where the cellulose fiber orientation is irrelevant.

#### 3.2.2. Optimization of Flow Channel Barrier Width 

**Figure 3:**
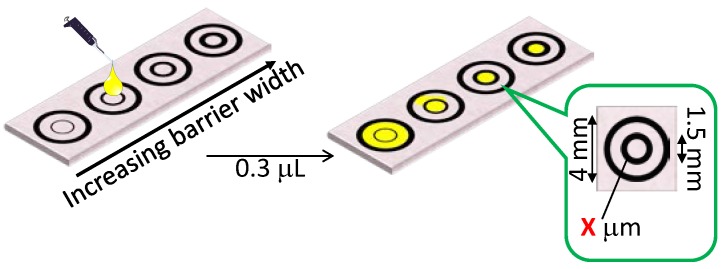
Schematic of the experimental method used to evaluate the minimally required wax barrier width. The indicated dimensions (*x* = 200–350 µm) refer to the values set for wax printing and do not represent the actual dimensions obtained after hot lamination.

One important factor determining the achievable spatial resolution of the microfluidic structure on a µPAD is the thickness of the hydrophobic barriers that is required to prevent the leaking of the transported sample liquid. Thinner barriers allow for higher densities of microfluidic channels, which contributes to an overall size reduction of devices and can therefore work with smaller sample volumes. The narrowest printed wax line widths resulting in reliable fluid barriers were experimentally determined. Leaking tests were performed similarly to a previously described method, as schematically outlined in Figure 3 [15]. Two concentric circles of wax with 1.5-mm and 4.0-mm diameters were printed on both sides of the filter paper and melted into the paper by hot lamination. While the line width of the outer circle was kept constant at 500 µm, the value of the inner circle was varied between 200–350 µm in increments of 50 µm.

A barrier is regarded as being functional, if no leaking of liquid from the inner circle occurs. The performance of a hydrophobic barrier depends on the applied sample volume per area. For this reason, the current experiment was performed with samples of larger volume per area compared to those encountered in a final device. Table 1 lists the number of functional barriers obtained among 20 repetitions. The minimally required barrier width resulting in a 100% success rate (20/20) was determined as 300 µm (value set for printing), corresponding to an actual barrier width of 467 ± 33 μm after passing through the hot laminator.

#### 3.2.3. Optimization of Flow Channel Width

**Figure 4:**
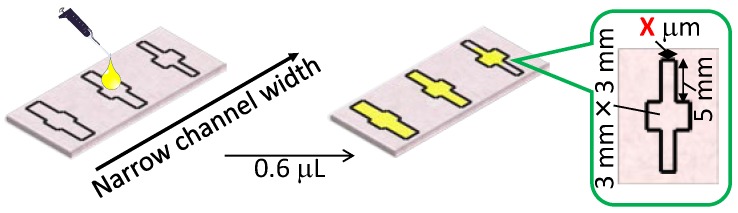
Schematic of the experimental method used to evaluate the minimally required width of microfluidic channels aligned to the cellulose fiber direction in the filter paper. The indicated dimensions refer to the values set for wax printing and do not represent the actual dimensions obtained after hot lamination.

The width and length of the microfluidic channels in a µPAD directly influence the minimal amount of sample liquid required to fully wet the device. While the channel lengths can be adapted to the needs arising from specific assays to be performed on a µPAD, their minimal width is determined by the requirement to achieve reliable fluid transport. To decide on the narrowest channel width for unhindered wicking of the sample, liquid flow with varying channel widths was experimentally evaluated as shown in Figure 4. Based on the results described above, flow channels were aligned in parallel to the cellulose fiber direction of the paper (MD) for this experiment and printed with 300-µm barrier line widths and channel widths of 300–500 µm on both sides of the filter paper.

A sample volume of 0.6 µL was selected to guarantee a liquid volume sufficient for complete wetting of the microfluidic paper structure with the widest channels evaluated. Table 2 summarizes the results of 20 independent trials for each channel width. As in the case of optimizing the hydrophobic barrier width, a flow channel was only regarded as reliable in the case of a 100% success rate (20/20). According to Table 2, 400 μm of printed channel width is the narrowest channel value to obtain unhindered sample transport. This printer set width results in actual microfluidic flow channels of 228 ± 30 μm after lamination, which is currently the narrowest value reported by any printing method for µPADs.

#### 3.2.4. Optimized µPAD Design

**Figure 5:**
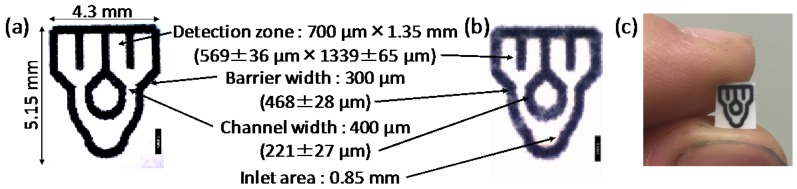
Micrographs of a wax-printed microfluidic paper-based analytical device (µPAD): (**a**) before and (**b**) after hot lamination. The scale bars correspond to a length of 1 mm. Dimensions are indicated before and after hot lamination (values in parentheses). (**c**) Corresponding photograph.

Based on the experimental results described above, the following conditions were considered to be important to obtain a µPAD, in which a single sub-microliter sample is reliably and reproducibly distributed into multiple detection zones through a network of microfluidic channels: (a) double-sided wax printing of aligned identical patterns; (b) a symmetric pattern design with all microfluidic channels aligned parallel to the cellulose fiber direction of the paper; and (c) identical flow distances from the sample inlet to all detection zones. One possible device design meeting these conditions is shown in Figure 5. It consists of four detection zones located at the end of microfluidic flow channels and one single sample inlet. The size of the detection zones has been selected to allow for the acquisition of colorimetric signals by conventional desktop scanners without having to refer to microscopic techniques.

The minimum amount of aqueous sample liquid required to fully wet all of the four detection zones of the µPAD was evaluated by applying 0.3–0.6 µL of colored aqueous solution. Table 3 lists the results of five independent trials for each respective liquid volume. The results demonstrate that a volume of 0.5 µL is sufficient to reliably fill all of the four detection zones. The experiment also reveals that lower sample volumes lead to incomplete wetting of detection zones with significant differences in flow distance. An optimized symmetric pattern design with identical lengths of flow channels cannot completely eliminate the influence of inherent spatial differences in a filter paper substrate in terms of cellulose fiber orientation and density. The present required liquid volume of 0.5 µL is to the best of our knowledge the lowest reported for a paper-based device with a single sample inlet and a patterned microfluidic structure terminating in multiple detection zones.

### 3.3. Human Serum Albumin (HSA) Assay

#### 3.3.1. Colorimetric Assay with Sub-Microliter Sample Volume

The colorimetric quantification of total protein content, with human serum albumin (HSA) as a representative protein, was used to demonstrate the analytical performance of an optimized µPAD design in a simple model assay. The detection relies on the pH-sensitive indicator tetrabromophenol blue (TBPB), which upon interaction with proteins at acidic pH undergoes a color change from yellow to blue [21]. A sample matrix simulating the composition of human tear fluid was selected, since it represents a type of sample typically only available at small volumes. The identical colorimetric reagent was deposited onto all of the four detection zones of the µPAD before lamination using a conventional desktop inkjet printer. In order to eliminate any issues caused by a potential misalignment of printed reagent ink, TBPB solution was printed in a continuous rectangular shape covering all detection zones. The chosen arrangement enables the simultaneous acquisition of quadruple data points from a single application of sample liquid. However, in the present model assay, it mainly serves the purpose of demonstrating the small interchannel variations obtained with a µPAD design optimized for small sample volumes. 

**Figure 6:**
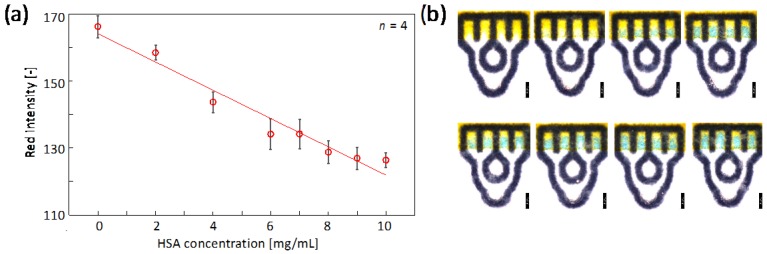
Colorimetric human serum albumin (HSA) analysis through the application of 0.8 µL of sample liquid to an optimized µPAD. (**a**) Calibration curve with data plots and error bars representing mean red intensities and corresponding standard deviations extracted from the four detection zones (parameters of regression line shown in Table 4); (**b**) Micrographs of µPADs after application of 0, 2, 4, 6, 7, 8, 9, and 10 mg/mL HSA (from upper left to lower right). The scale bars correspond to a length of 1 mm (brightness and contrast adjusted for improved visibility).

Figure 6a shows a calibration curve obtained for HSA after the single application of 0.8 µL of sample liquid. A separate µPAD was used for each single HSA concentration. The linear range of 0–10 mg/mL corresponds to typical total protein concentrations found in human tear fluid [22]. Figure 6b shows a series of micrographs of the corresponding µPADs. It should be noted, however, that the colorimetric data analysis has been performed on standard color scanned images without the use of a microscope.

The mean of the relative standard deviations for the colorimetric signal intensity in the four detection zones was calculated as 2.40% (Table 4). This value indicates a reasonably low interchannel variation in sample flow passing through the parallel microfluidic channels.

#### 3.3.2. Sample Volume Variation Tolerance of the Colorimetric Signal

**Figure 7:**
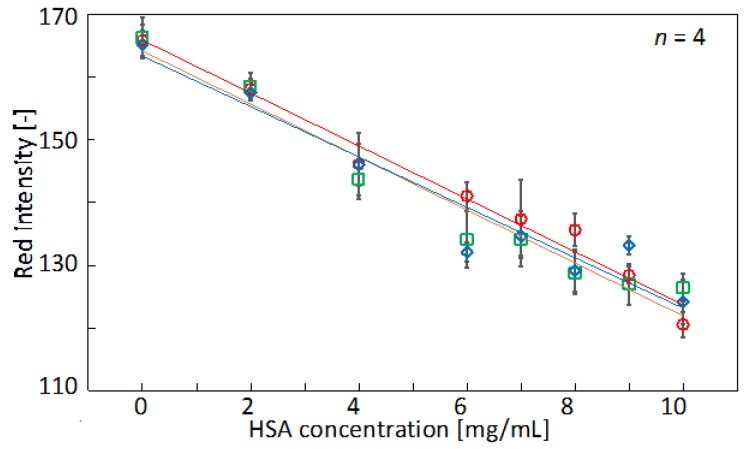
Calibration curves for HSA obtained with variable sample volumes: 0.7 µL (red circles), 0.8 µL (green squares), and 0.9 µL (blue diamonds). Data plots and error bars represent mean red intensities and corresponding standard deviations extracted from four detection zones (parameters of regression lines shown in Table 4).

Colorimetric signals obtained in assays performed on µPADs are generally dependent on the volume of the applied sample liquid. The colorimetric signal is determined by the absolute amount of analyte transported through the cellulose fiber network to the colorimetric detection zone, rather than the concentration of the sample alone. When working with sub-microliter samples, the risk of pipetting-related variations in sample volume inevitably increases. On the other hand, it has been anticipated that the closed space created by full lamination of the µPAD used in the current study contributes to controlling the sample volume that can be absorbed by the device. In order to evaluate the tolerance against small variations in the applied sample liquid volume (±0.1 µL), HSA response curves were recorded between 0.7–0.9 µL of applied sample. The resulting data is summarized in Table 4 and Figure 7.

The results demonstrate an acceptable tolerance against minor variations in the applied sample volume, since slopes and intercepts of the calibration curve are not significantly different. However, despite full lamination of the µPADs, the colorimetric signal does remain dependent on the amount of applied sample, and total volume independence is not achieved. It has to be kept in mind that paper is a rather heterogeneous material and that, therefore, variations in liquid volumes adsorbed by the cellulose fiber network are inevitable.

## 4. Conclusions 

The current study has demonstrated the possibility of using the popular wax-printing technology combined with the most widely used filter paper substrate to obtain miniaturized paper-based analytical devices with microfluidic structures, enabling the analysis of samples only available in small volumes. To reach this goal, the consideration of several factors influencing the resolution of the microfluidic patterning process was necessary. It has been experimentally demonstrated that replacing the hot plate by simple hot lamination as a heating method for melting printed wax into the paper substrate is a key factor in achieving increased patterning resolution. This approach does not significantly alter the costs for device fabrication and is expected to be fully compatible with roll-to-roll mass-production methods. The device presented is just one possible model. It is clear that other factors not evaluated here, such as the length of microfluidic channels or the integration of additional reaction zones required to perform a specific assay, are essential in determining the minimal volume of sample required in a µPAD. However, we believe that the current work demonstrates that the applicability of µPADs is not limited to samples available in amounts of several microliters, but can be extended into the sub-microliter volume range.

## Figures and Tables

**Table 1:** Evaluation of minimally required printed wax barrier width (images converted to grayscale and contrast adjusted for improved visibility).

Printer Set Wax Barrier Width (µm)	200	250	300	350
Number of functional barriers (*n* = 20)	13/20	12/20	20/20	20/20
Actual pictures	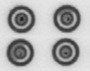	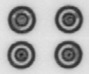	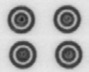	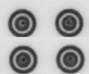

**Table 2:** Evaluation of narrowest possible printed microfluidic channel width (images converted to grayscale and contrast adjusted for improved visibility).

Printer Set Channel Width (µm)	300	400	500
Number of functional channels (*n* = 20)	4/20	20/20	20/20
Actual pictures	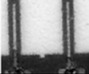	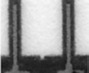	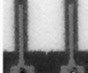

**Table 3:** Evaluation of the minimal sample volume required to completely fill the model µPAD with multiple detection zones; the scale bars correspond to a length of 1 mm (images converted to grayscale and contrast adjusted for improved visibility).

Sample Volume (µL)	0.3	0.4	0.5	0.6
Success rate (*n* = 5)	0/5	1/5	5/5	5/5
Actual pictures	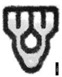	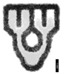	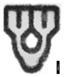	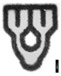

**Table 4:** Slope and y-intercept values of linear HSA response curves (0–10 mg/mL) recorded by application of variable sample volumes.

Sample Volume (µL)	Slope	*y*-Intercept	*R*^2^	Mean of Relative Standard Deviations
0.7	−4.21	166	0.979	2.08**%**
0.8	−4.22	164	0.956	2.40**%**
0.9	−4.01	163	0.931	1.78**%**
Average	−4.15 ± 0.12	164 ± 1.5	-	-

## Supplementary Materials

The following are available online at http://www.mdpi.com/2072-666X/7/5/80/s1: Figure S1: Heating time dependent widths of wax lines measured after hot plate (150 °C) treatment under various conditions for untreated and compressed filter paper. Figure S2: Thickness of filter paper before and after passing the hot laminator. Figure S3: Cellulose fiber orientation-dependent sample wicking velocities.
