# Supplementary material for: High-Resolution Microfluidic Paper-Based Analytical Devices for Sub-Microliter Sample Analysis
Source: Micromachines (Basel). 2016 May 2;7(5):80. doi: 10.3390/mi7050080 (PMC6189915; doi:10.3390/mi7050080)
Supplement: Supplementary file 1 [file micromachines-07-00080-s001.pdf]

Supplementary Materials: High-Resolution Microfluidic Paper-Based Analytical Devices for Sub-Microliter Sample Analysis

Keisuke Tenda, Riki Ota, Kentaro Yamada, Terence G. Henares, Koji Suzuki and Daniel Citterio

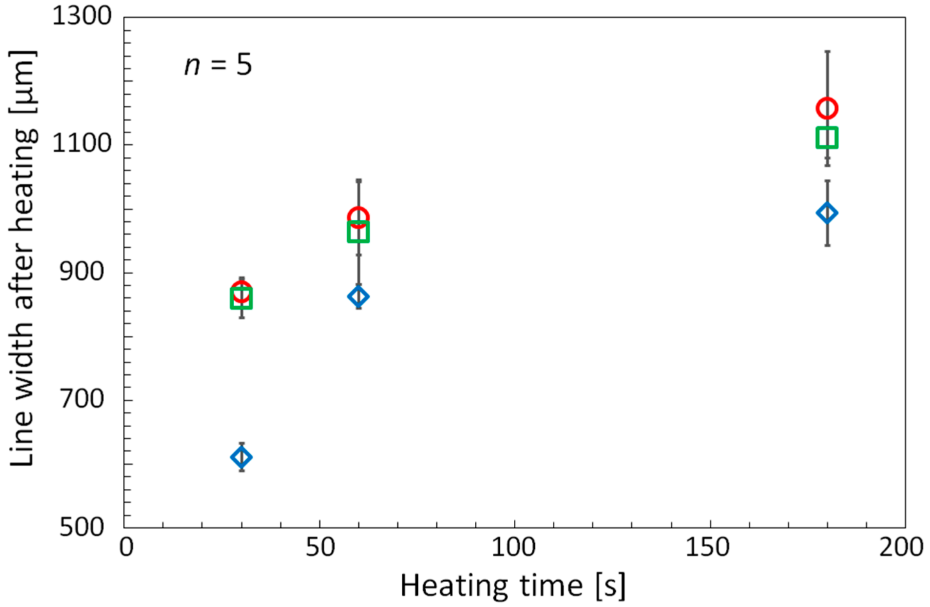

Figure S1. Heating time dependent widths of wax lines (original printing width: 300 µm) measured after hot plate (150 °C) treatment under various conditions: wax line printed on untreated Whatman 1 filter paper (red circles) (“standard” hot plate procedure); wax line printed on untreated Whatman 1 filter paper with uniform weight (22 kg) applied during heating (green squares); wax line printed on compressed Whatman 1 filter paper immediately after passing the paper through the laminator for compression (blue diamonds) (for this purpose, a sheet of filter paper was sandwiched between two laminate films, while being protected with sheets of cooking paper on both sides, to prevent the actual lamination process).

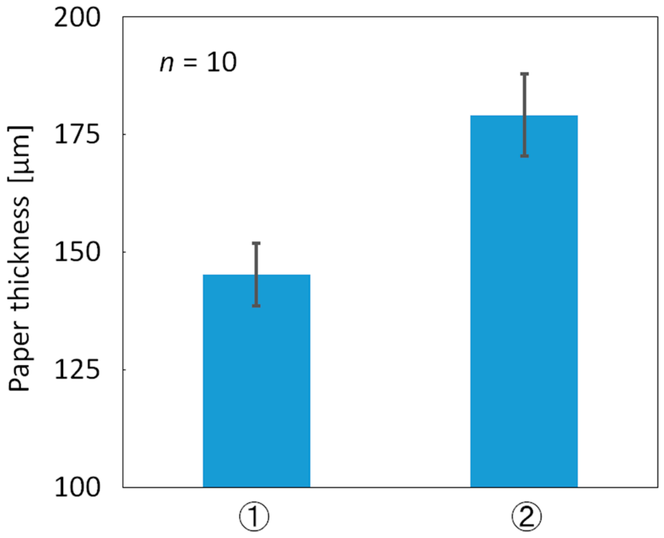

Figure S2. Thickness of Whatman 1 filter paper; ① after passing through the hot laminator (while preventing the actual lamination process as described in the caption to Figure S1); ② without pressure application (untreated filter paper).

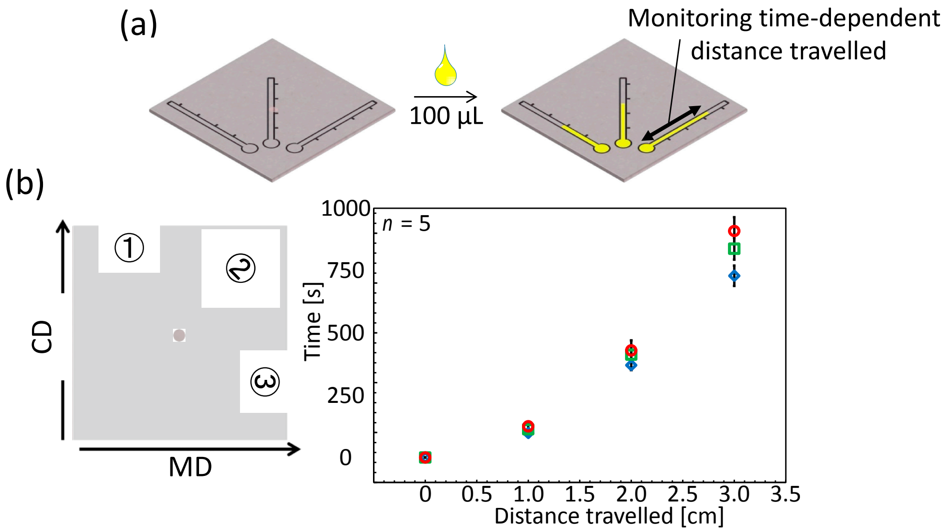

Figure S3. (**a**) Schematic representation of the evaluation of the influence of cellulose fiber direction on sample wicking velocity in single filter paper channels (channel width: 632 ± 36 µm (n = 20) after lamination). The flow distances are measured as indicated by the arrow. (**b**) Quantitative results averaged for 5 independently fabricated devices (mean value ± 1σ). Red circles, green squares, and blue diamonds in the graph represent the flow directions ①, ②, and ③ shown in the left scheme, respectively.

Figure S3b clearly demonstrates that faster flow velocities are achieved in microfluidic channels aligned to the machine direction (③) (blue diamonds) with parallel orientation of the cellulose fibers, compared to the cross direction (①) (red circles), where flow occurs perpendicular to the cellulose fiber orientation. Channels arranged in a 45° angle to machine direction (MD) or perpendicular cross direction (CD) (② in Figure S3b) (green squares) show flow velocities approximately between the two extremes. These results confirm that the cellulose fiber direction in the paper substrate does affect sample flow velocity in a single-channel device.

The laminated devices developed in this work do not show accelerated sample liquid flow, unlike a previous report for PET film-covered paper-based microfluidic channels [1]. On the contrary, sample flow is generally slow in the fully laminated devices. Decreased sample flow rates are attributed to the smaller pore size of the compressed filter paper and the fact that there is no gap between the laminate film and the paper substrate. In the case of the PET film-covered devices, where much faster liquid transportations are achieved, an 810-µm gap exists between the paper and the film cover. In such a situation, sample liquid is transported quickly via the capillary force, but this is not the case in the current laminated devices, where sample flow is restricted to the cellulosic network, due to the absence of an air gap.

Reference

1. Anbuhi, S.J.; Chavan, P.; Sicard, C.; Leung, V.; Hossain, S.M.Z.; Pelton, R.; Brennan, J.D.; Filipe, C.D.M. Creating fast flow channels in paper fluidic devices to control timing of sequential reactions. *Lab Chip* **2012**, *12*, 5079–5085.

© 2016 by the authors; licensee MDPI, Basel, Switzerland. This article is an open access article distributed under the terms and conditions of the Creative Commons by Attribution (CC-BY) license (http://creativecommons.org/licenses/by/4.0/).
